# Annotations

**Published:** 1907-10-05

**Authors:** 


### ANNOTATIONS.

### Morphine and Retention of Urine.

In a recent clinical lecture Sir Wm. Bennett relates an interesting experience which may be usefully noted for practical application. The lecture, among other aspects of difficult micturition, related to those cases in which, though the urine either is not or cannot be voided, a catheter of full size is readily passed into the bladder. To illustrate a possible cause of this condition Sir William quotes the case of a young man who at occasional intervals suffered from retention of urine, compelling the use of a catheter. This condition had brought him under the notice of many medical men, but no effective interpretation or solution of the problem had been found. A remarkable feature of the case was the fact that the patient appeared to think very little of the matter, and was almost or altogether free from anxiety and distress—this being, of course, in marked contrast to the acute alarm which inability to pass water usually excites in the ordinary individual. In the course of examination, however, it was observed that on the thighs were a number of spots suggestive of the bites of some insect and similar spots, some indurated, were present on the forearms. For these, as is invariable in such cases, the patient had an explanation in the shape of an "irritable skin." But it was manifest to the experienced eye that the marks found on his limbs were produced by a hypodermic needle, and thus the conclusion was inevitable that the patient was a victim of the morphine habit, and that the recurring retention of urine depended on an occasional excessive dose of the drug. The case illustrates the necessity of the "detective method" in clinical work, more especially where there is a mystery to be cleared away.

### Lectures on Old Age.

The programme for the ensuing session of the London Polyclinic includes a series of lectures on old age in its medical relations which will doubtless excite considerable interest. Much attention has of recent years been paid to the preservation of life in its earlier years, and at times books and lectures have shown that the characters of disease in the declining stages of existence are well worthy of attention. There are, in other words, clinical peculiarities both at one and the other extreme of human life, and practical claims not less than scientific curiosity demand attention to these. In the Polyclinic lectures it will be recognised that a very successful attempt has been made to deal adequately with the various aspects of disease in advanced life, and, indeed, the institution is to be congratulated upon its ability to present so attractive and authoritative a list. The opening lecture of the series is by Dr. F. W. Hewitt and the subject is, "The Administration of Anæsthetics to the Aged"; then in due course follow Dr. Savage on "The Psychoses of the Aged," Sir Wm. Gowers on "The Nervous System in Old Age," Professor Clifford Allbutt on "The Senile Cardio-Vascular System," Mr. Marcus Gunn on "The Eyesight in Old Age," Sir Richard Douglas Powell on "Senile Respiratory Disorders," Dr. Burney Yeo on "Diet in Old Age," and Dr. Radcliffe Crocker on "The Skin-troubles of the Aged." This is, indeed, a remarkable programme, and, it can hardly be doubted, will make a wide appeal. Another feature of the Polyclinic programme which seems worthy of recognition is its inclusion of representatives from the Scottish, Irish, and provincial schools. In the forthcoming session quite a number of the lecturers are drawn from these schools and to the great advantage we have no doubt of their audiences. Those engaged here in post-graduation study will welcome the chance of hearing men whose work has done much, not only for the interests of the schools with which they are specially associated, but also for the general advance of medicine.

### More Poisonous "Tabloids."

In our issue of August 31 we drew attention to a police prosecution of a limited company for the sale of "tabloids" containing strychnine without satisfaction of the conditions demanded by the provisions of the Pharmacy Act. On the occasion in question it was urged in defence that, even if a technical offence had been committed, the practice was one generally adopted by chemists and druggists. An attempt was made to submit evidence in support of this position, but the magistrate held that such evidence was irrelevant, and in imposing a substantial fine he remarked that if the practice were a common custom it was well to mark the offence as one of considerable gravity. The defendants in the case have now managed to justify their contention that in selling "tabloids" containing strychnine they were not greater sinners than their neighbours, and in doing so have brought to book no less a person than the ex-President of the Pharmaceutical Society. Having through an agent made purchases of "tabloids of Easton's syrup" from various chemists in London they discovered that many of the vendors were in the habit of handing such "tabloids" across the counter to any chance purchaser without any of the formalities insisted on by the law in reference to the sale of strychnine and other substances scheduled as "poisons" in the Pharmacy Act. They, therefore, laid an information against one of these offenders who, in consequence, had to appear at the Westminster Police Court, where he offered "a technical plea of guilty" and submitted to a fine of £9 and costs. The neglect was a particularly marked one, and involved a breach of the law in three respects. The bottle containing the "tabloids" was not labelled with the name and address of the seller, the purchaser was not known to the seller, and no entry of the sale was made in the "Poisons Book." From all this it is evident that a very loose custom has grown up among pharmacists, and it is satisfactory in the public interest that the law has been vindicated. With the motives that have prompted these prosecutions we have no concern, but we repeat that a large responsibility falls upon all who are concerned in the ready distribution of poisonous drugs to the public in the seductive form of compressed tablets. If these are sold at all, their sale should certainly be conducted under conditions which impress the purchaser with the risk he is undertaking.

